# Virtual Med-Peds: Description of the First Virtual Med-Peds Student Elective During COVID-19

**DOI:** 10.7759/cureus.11971

**Published:** 2020-12-08

**Authors:** Himani Divatia, Allen R Friedland

**Affiliations:** 1 Pediatrics, ChristianaCare, Newark, USA

**Keywords:** medical student electives, virtual curriculum, covid-19, med-peds, residency application process, mentorship, undergraduate medical education

## Abstract

Background: For medical students seeking additional specialty experience in Med-Peds, in-person electives have often been a source of mentorship and guidance. The COVID-19 pandemic has impacted the ability for the completion of in-person clerkships for medical students across the nation. Virtual opportunities to increase exposure to Med-Peds programs and didactics are lacking at this time.

Objective: To develop a virtual Med-Peds student elective that serves to increase awareness of the Med-Peds specialty, exposure to Med-Peds topics and relevant didactics, and exposure to Med-Peds specific mentorship when on-site clerkships are not available due to the COVID-19 pandemic.

Methods: Fifteen medical students participated in a virtual Med-Peds student elective utilizing Zoom (Zoom Inc, San Jose, CA). Three separate cohorts of five students each completed two-week elective experiences. The virtual elective curriculum was created using asynchronous and synchronous learning modalities. Sessions were composed of self-directed learning topics, peer-to-peer interactive case discussions, resident-led didactics, and attending physician-led didactics and mentorship sessions. A pre-survey was administered at the beginning of the elective and a post-survey was administered at the end of the elective to assess the effectiveness of the elective, student experiences with Med-Peds mentors, and students’ general perceptions of Med-Peds as a residency application choice.

Results: All students (100%), rated the Med-Peds elective to have exceeded their expectations. All students indicated this elective had been extremely (100%) valuable to increase their understanding and interest in Med-Peds (top rating on a five-point Likert scale). Compared to prior to the elective, most were very likely (87%) or likely (7%) to apply to Med-Peds as their top (preferred) specialty. Similar to pre-survey data, one-third (33%) of the students were still likely to apply to an alternate specialty in addition to Med-Peds. Hundred percent of students indicated that the mentorship component of the elective exceeded their original expectations. While most students indicated that they are much more strongly considering applying to Med-Peds as a top (preferred) specialty, the number of students who continue to consider dual-application to include either categorical Internal Medicine, categorical Pediatrics, or Family Medicine did not differ before and after completion of the virtual elective.

Conclusions: Implementation of a virtual medical student elective focusing on exposure to Med-Peds can strengthen medical students’ interest in the combined specialty despite a paucity of previous experiences or an affiliated Med-Peds program. This new type of rotation can positively impact a student’s view of a hospital system and a residency program when in-person clinical rotations are not available.

## Introduction

In light of the COVID-19 pandemic, medical students’ exposure to direct patient care has been significantly limited. As guided by the Association of American Medical Colleges (AAMC), clinical rotations for medical students were suspended amidst the pandemic first surge [1]. Mandatory core rotations that serve to inform decisions regarding specialty choice interest were either postponed or never completed during a critical window in the lead up to the application cycle for the 2021 match [2]. Thus, medical students missed an opportunity to gain increased exposure to specialties they are strongly considering applying to in the 2021 residency application cycle. 

For medical students interested in applying to combined Internal Medicine-Pediatrics (Med-Peds), rotation changes are thought to have detrimental impacts on student interest in the field. Med-Peds requires an interest in two specialties, Internal Medicine and Pediatrics, and many medical students have not completed both third-year clerkships by the time of the beginning of their official fourth year. Many students still experience difficulty completing third-year clerkships and obtaining important sub-internship (sub-I) rotations early in their fourth-year schedule to close educational gaps, decide on residency specialty choices, and obtain letters of recommendation. Up to 200 students per year rely on specific Med-Peds fourth-year rotations to provide a deeper understanding and experiential knowledge of the Med-Peds specialty [3]. Furthermore, the elective rotations serve as a platform for building a mentorship with Med-Peds trained faculty and advisors and provide a direct view of the structure and culture of a residency program [4]. However, away rotations have been discouraged by many national educational organizations even as restrictions eased in order to adhere to social distancing best practices. This has left a large proportion of students without direct exposure to the combined specialty.

We developed a series of two-week virtual Med-Peds student electives, not for credit, to help close the gap for third-year students not on any clinical rotation and without much exposure or mentorship to Med-Peds. The objectives of the virtual elective were to enable students to understand the combined specialty of Med-Peds in general, to explore the kinds of career paths Med-Peds physicians pursue, and to learn topics (adult and pediatric) germane to the practicing Med-Peds physician. At the time of this publication, this is the only known virtual Med-Peds student elective amongst the 77 Med-Peds residency programs. This study will describe the structure and outcomes of this rotation.

## Materials and methods

Participant selection

Medical students were invited electronically based on their application already received to our in-person Med-Peds fourth-year elective at ChristianaCare. These students were unable to be offered an in-person rotation due to pandemic restrictions and the inability for student schedules’ to coincide with in-person elective opportunities later in the academic year. A total of twenty medical students were offered with five students that declined or did not respond to our offer. A total of fifteen medical students agreed to participate in the virtual elective.

Participant characteristics

All students were not on any clinical rotation. Fourteen students were from medical schools not affiliated with any Med-Peds residency program. Four students had not completed their Internal Medicine clerkship and two students had not completed their Pediatrics core clerkship prior to the virtual elective. Five students had prior interactions with Med-Peds residents for specialty advice, three students had experiences with Med-Peds faculty, four students had received mentorship from a Med-Peds program leader, and three students had no Med-Peds trained mentors. All fifteen students had varied experiences with mentors trained in a specialty other than Med-Peds but did not have a common “go to” person type to learn about Med-Peds. Table 1 demonstrates baseline data of the students who participated in the virtual elective. 

**Table 1:** Baseline Characteristics of Medical Student Participants

	n = 15
D.O. candidate	12 (80%)
M.D. candidate	3 (20%)
Medical school region	
Northeast	8
Midwest	3
South	2
West	2
Gender	
Male	5
Female	10
Prefer to self-describe	
Med-Peds trained mentors for specialty advice	
Med-Peds faculty	3
Med-Peds resident	5
Med-Peds Program Director or Associate Program Director	4
No Med-Peds mentor	3
Non Med-Peds trained mentors for specialty advice	
Dean	3
Internal Medicine faculty	4
Internal Medicine Chair	2
Pediatric faculty	4
Pediatric Chair	3
Other	3

Materials

Zoom (Zoom Inc, San Jose, CA) was utilized as the platform for the virtual Med-Peds student elective. A daily Zoom “room” was created for participants and presenters to log into utilizing a meeting ID and passcode. Students were expected to use a webcam, computer audio, and the chat feature. A separate google drive was created to serve as a repository for all documents relevant to the elective.

Curriculum design

The ChristianaCare Virtual Med-Peds student elective was designed as a two-week interactive experience utilizing the virtual platform Zoom for delivery of didactic content as well as interactive experiences. Three separate cohorts, each for two weeks, consisting of five medical students per cohort participated during May and June 2020. 

The curriculum had six different components, emphasizing concepts of adult learning theory. The content was available for both synchronous and asynchronous learning. Core components of the curriculum included (1) daily self-directed learning times encouraged to increase exposure to a variety of evidence-based guidelines and relevant literature; (2) peer to peer (student) interactive case-based sessions as well as podcast shares to promote motivation for learning and team building amongst the cohort; (3) Med-Peds resident-driven sessions focused on case discussions, NEJM Knowledge+ interactive patient cases, resident scholarly activity, and discussions on residency planning, wellness, and mentorship; (4) Med-Peds attending sessions, with a mixture of ChristianaCare and non-ChristianaCare trained attendings. Topics were based on their own specialized clinical and professional interests and expertise; (5) cases, didactics, and readings for content; and (6) reflective writing assignment at the end of the elective that focused on their experience, understanding of Med-Peds, and whether or not their goals prior to starting the elective had been met.

The schedule was generally from 9 am to 5 pm, and most individual sessions were up to one hour in duration. All students had the lunch hour as protected time off and breaks were scattered throughout the day. Topics were chosen based on medicine and pediatric core curriculum highlighted by the American Board of Internal Medicine (ABIM) and American Board of Pediatrics (ABP) blueprints [5,6]. All students received two group sessions on leadership development and general Med-Peds specialty statistics and one session per week for debriefing and feedback pertaining to the virtual elective. Each student was also offered one hour of individualized mentorship with the program director and associate program director, if desired. All fifteen students accepted these individualized mentorship sessions. No medical school credit or letters of recommendations were provided. A certificate of completion was given to each participant.

On average, each cohort received seven sessions of self-directed learning, six sessions of peer group learning, 10 sessions of resident-led learning, and 20 sessions of attending physician-led learning. A sample schedule is shown below in Table 2.

**Table 2:** Med-Peds Virtual Student Elective Sample Schedule Green: self-directed learning; Pink: peer to peer sessions; Yellow: resident-led sessions; Blue: Med-Peds faculty-led session

	WEEK 1							WEEK 2		
9-10 AM		Self-Directed Learning - Milestones	Self-Directed Learning - VTE Prophylaxis	Self-Directed Learning - Diabetic Preventive Care	Self-Directed Learning - Dysphagia		Self- Directed Learning- Infectious Endocarditis	Sickle Cell and AYA Oncology	Self-Directed Learning - Pediatric Platelet Disorders	Self- Directed Learning - Juvenile Idiopathic Arthritis
10-11 AM	Orientation/ Onboarding		Peer to Peer - Podcast Share	Communication in Palliative Care	Applying to Residency: ERAS, Interviewing, Match		Leadership	Clinical Case Review	Evidenced-Based Medicine Journal Club	Evidenced-Based Medicine Journal Club
11-12 PM	Pediatric PNA	Peer to Peer: NEJM interactive case	Transition and CP Through the Ages	Pediatric UTIs	Scholarly Activities During Your M4 Year; How to Write an Abstract		Global Health	Clinical and Diagnostic Reasoning	Peer to Peer - NEJM Interactive Case	Firearm Violence Prevention for Every Clinician
12- 1 PM	Lunch	Lunch	Lunch	Lunch	Lunch		Lunch	Lunch	Lunch	Lunch
1-2 PM	Cognitive Bias	Immunizations	Social Determinants of Health/ACES	Resiliency and Self Care	Global Health, National Leadership, PHM potpourri		EKG review	Peer to Peer - podcast share	Lifestyle Medicine- physical activity, sleep, mental/emotional well-being	Week 2 Check-In/Debrief
2-3 PM	Chief Resident Welcome	What’s new in Telemedicine	Overview of Common Pediatric and Adult Cardiac Conditions and Their Management				Med-Peds Zebras	Dermatology Pearls for the Generalist	Patient Safety	Reflective Writing
3-4 PM	Acute Renal Failure	Welcome to Delaware	Delivery Room/NRP	Integrative Modalities in Residency and Practice	Week 1 Check-In/Debrief		Intro to Culinary Medicine	Peer to Peer - Podcast Share	Sepsis	
4-5 PM								Pediatric Pulmonology Pearls		
6-8 PM			Med Peds Meeting 6-8							

Study

An anonymous 11-question pre-survey and 16-questions post-survey was administered prior to and at the completion of the elective, respectively, via SurveyMonkey. This study was deemed exempt by the ChristianaCare institutional IRB as it was conducted in an educational setting using normal educational practices. 

## Results

All students (100%) from all three cohorts completed both pre and post-surveys.

Pre-survey

Students indicated that gaining mentorship within Med-Peds, experiences with different Med-Peds people, program-specific information, and exposure to different job-niches of Med-Peds were extremely important for interest in the virtual elective.

Overall

All students (100%), rated the Med-Peds elective to have exceeded their expectations. All would recommend the ChristianaCare Virtual Med-Peds student elective to another student in the future and all think more positively about the residency program.

Elective construct

The students were surveyed about aspects of the elective and are listed in the table below. All participants were overall very satisfied or satisfied with the virtual platform. Fourteen of 15 students submitted their most memorable takeaway and are categorized below in Table 3.

**Table 3:** Most Memorable Takeaways from Elective

Med-Peds Culture	
	Learning how Med-Peds physicians think about the same topic in different ways
	Learn how to manage complex diseases through the whole age spectrum
	Med-Peds is about passion for medicine and finding ways to express that based on personal interest
	Versatile career with passionate people
	Family feel and amazing culture of Med-Peds people
Med-Peds Careers	
	Variety of career pathways and diverse scope of practice
	Med-Peds career paths are what you make of them, all doors open
	Uniquely situated to care for the transition population, dual training gives unique perspective in comparison to categorical counterparts
	Role of Med-Peds in global health and advocacy
Med-Peds Camaraderie	
	Interaction with the residents
	Meeting peers in each cohort who share similar interests and passions
	Camaraderie among Med-Peds physicians
Med-Peds Mentorship	
	Mentorship and valuable advice
	Case presentations and education delivered

Fourteen of 15 students submitted their least favorite aspect of the elective, of which four responded that they had no least favorite aspect. The other 10 students’ comments are categorized below in Table 4.

**Table 4:** Least Favorite Aspects of Elective

Elective Duration	
	That it was only two weeks in duration, wished it could have been longer
	Sometimes the days were a little long, especially if there was a night session
	Sitting for so long
Elective Construct	
	That it was virtual, and not in person
	Difficult to get a sense of what resident life at ChristianaCare feels like
	Missed being able to experience the people and the facilities in-person
Elective Schedule	
	Unpredictability with scheduling, but understandable given the circumstances
	At times, lectures and discussions were cut short due to tightly packed schedule
	Not enough time with other students, and would like more time with new interns

Specialty and student impact

All students indicated this elective had been extremely (100%) valuable to increase their understanding and interest in Med-Peds (top rating on a five-point Likert scale). Students rated the impact of the elective on their decision to apply to Med-Peds and to other residency program types, as depicted in Table 5 below.

**Table 5:** Impact of Elective on Choice of Specialty for Residency Application

	Strongly Agree	Agree	Neutral	Disagree	Strongly Disagree
Elective strengthened my decision to apply to Med-Peds	14 (93.3%)	1 (6.7%)	0	0	0
Elective strengthened my decision to apply to another residency program type	1 (6.7%)	0	0	9 (60%)	5 (33.3%)

Compared to prior to the elective, most were very likely (87%) or likely (7%) to apply to Med-Peds as their top (preferred) specialty. However, compared to prior to the elective, two-thirds of students are very likely (33%) or likely (33%%) to apply to Med-Peds as their only specialty which is similar to pre-survey.

Alternate specialties (students were not allowed to choose more than one specialty) considered by students are in Table 6. Reasons for applying to programs in addition to Med-Peds are listed in Table 7.

**Table 6:** Alternate Specialties Considered by Med-Peds Virtual Elective Students

Categorical Internal Medicine	8
Categorical Pediatrics	2
Family Medicine	1

**Table 7:** Factors Contributing to Decision-Making for Dual Application to Med-Peds and an Alternate Specialty

Medical School Associated Factors	
	“There is a lot of pressure from my school to apply to another specialty other than Med-Peds”
	My dean and career advisor advised me to apply to another specialty in addition to Med-Peds to ensure that I will match as med-peds is more competitive
	In light of the different application year and that fact that my Peds clerkship has been moved to the fourth year, I have been encouraged by my dean and advisor to apply to IM programs as a backup
	school’s suggestion is that we dual apply
Individual Factors	
	Location restrictions
	I have not had enough exposure to Med-Peds to make an informed decision. I’m hoping that this elective will help me make my decision
	Unsure about going into the application cycle as a D.O.
	Competitiveness as an applicant
Specialty Associated Factors	
	Inability to secure an in-person rotation in med-peds to express interest in the specialty
	There are more categorical medicine programs that emphasize my career interests in health equity and social justice
	To increase the chances of matching given the smaller percentage of Med-Peds residencies

All students (100%) rated the Med-Peds elective and mentorship to have exceeded their expectations. Direct feedback on the degree of mentorship they received, including the following comments:

“I did not expect to get so much useful advice about the application process in this elective. It also helped solidify the real focus that exists in Med-Peds for mentoring trainees at all levels.”

“To this point, I have not had a strong relationship with a mentor, but this rotation showed me how important and impactful it can be to have a good mentor.”

“Overwhelming support from the APD and PD who provided listening ears and advice that was specific to my concerns and needs.”

“I wasn’t sure what to expect beforehand, but it was an excellent experience that showed me the program feel. There were so many mentors that would give open and honest answers, open to all questions.” 

## Discussion

In the midst of the COVID-19 pandemic where the cessation of clinical experiences was required, the ChristianaCare Virtual Med-Peds student elective rotation provided a direct insight into the combined specialty through a virtual platform. The overall feedback from the rotation was highly positive, and students found the experience to be extremely valuable. The virtual platform was viewed favorably, and overall students felt that they had a strong sense of connection with the peers in their respective cohorts, as well as the program faculty and residents they encountered, with all students indicating that they would strongly recommend this elective rotation to future applicants.

While most students indicated that they are much more strongly considering applying to Med-Peds as a top (preferred) specialty, the number of students who continue to consider dual-application to include either categorical Internal Medicine, categorical Pediatrics, or Family Medicine did not differ before and after completion of the virtual elective. The barriers students indicated were external to what a virtual elective was intended to overcome, and included personal reasons such as geographic limitations, concerns about competitiveness to include being an osteopathic candidate, and advice to dual-apply. Although the virtual elective did not decrease the number of individuals considering dual-application, it is plausible that without an experience as such, fewer students would consider applying to Med-Peds as a top specialty and/or consider applying to a lower ratio of Med-Peds programs to categorical programs. Further follow-up after the residency match will be valuable in identifying the number of students from this elective who applied and matched into med-peds and what impacted their final rank lists. 

A highly valuable part of the elective which exceeded expectations for participants was career mentorship in Med-Peds. While all students had exposure to Med-Peds mentors to varying degrees, the majority of the students were receiving most of their career advice from non-Med-Peds trained physicians. The elective provided an opportunity to receive individualized mentorship in addition to general career advising from various Med-Peds physicians at all levels of training and practice. All students indicated that this was among the most important takeaway from the elective, and felt this highlighted the general culture and camaraderie that exists within Med-Peds trained providers. Further work to be explored includes connecting the local Med-Peds mentors with medical school deans and career counselors in order to increase general awareness of Med-Peds and the diverse careers possible within this unique specialty.

Overall, 100% of students strongly agreed or agreed that the elective rotation strengthened their decision to apply to Med-Peds. At the same time, 93% (14/15) of students disagreed or strongly disagreed that the elective strengthened their decision to apply to another residency program type.

Limitations of this virtual Med-Peds student elective includes a small sample size of fifteen students in addition to a single program experience. There was no comparison to a control group or to an in-person med-peds elective. All presenters were currently affiliated with the same institution which can limit students’ generalizability to the specialty as a whole. We also did not assess the relative competitiveness of students in the elective to confirm or refute the common concern about a students’ competitiveness. 

## Conclusions

Implementation of a virtual medical student elective rotation focusing on exposure to Med-Peds can strengthen medical students’ interest in the combined specialty despite a paucity of previous experiences or an affiliated Med-Peds program. This new type of rotation can positively impact a student’s view of a hospital system and a residency program when in-person clinical rotations are not available. More specialty-specific mentorship directed to students and to those that generally advise medical students about the competitiveness of the field is needed. Expansion of this elective to other times of the year or even when in-person rotations resume should be considered. A national Med-Peds virtual elective can be developed using the framework provided from this small study specifically targeting students without a nearby Med-Peds program.
